# *Faecalibacterium* Gut Colonization Is Accelerated by Presence of Older Siblings

**DOI:** 10.1128/mSphere.00448-17

**Published:** 2017-11-29

**Authors:** Martin Frederik Laursen, Rikke Pilmann Laursen, Anni Larnkjær, Christian Mølgaard, Kim F. Michaelsen, Hanne Frøkiær, Martin Iain Bahl, Tine Rask Licht

**Affiliations:** a National Food Institute, Technical University of Denmark, Lyngby, Denmark; b Department of Nutrition, Exercise and Sports, University of Copenhagen, Frederiksberg, Denmark; c Department of Veterinary and Animal Sciences, University of Copenhagen, Frederiksberg, Copenhagen, Denmark; University of Wisconsin—Madison

**Keywords:** *Faecalibacterium*, infancy, siblings

## Abstract

*Faecalibacterium prausnitzii* is a highly abundant human gut microbe in healthy individuals, but it is present at reduced levels in individuals with gastrointestinal inflammatory diseases. It has therefore been suggested to constitute a marker of a healthy gut and is associated with anti-inflammatory properties. However, factors affecting the colonization of *F. prausnitzii* in the human gut during early life are very poorly understood. By analysis of 16S rRNA amplicon sequencing data from three separate infant study populations, we determined the colonization dynamics of *Faecalibacterium* and factors affecting its establishment in the gut. We found that in particular, the presence of older siblings was consistently associated with *Faecalibacterium* gut colonization during late infancy and conclude that acquisition of *Faecalibacterium* is very likely to be accelerated through transfer between siblings.

**IMPORTANCE** *Faecalibacterium prausnitzii* has been suggested to constitute a key marker of a healthy gut, yet the factors shaping the colonization of this highly oxygen-sensitive, non-spore-forming species in the intestinal environment remain poorly understood. Here, we provide evidence from three separate infant study populations that *F. prausnitzii* colonization in the gut happens during late infancy and is affected by the number of older siblings in the family. We conclude that *Faecalibacterium* acquisition is highly likely to be accelerated by contact between siblings. Bearing in mind the immunoregulatory properties of *F. prausnitzii* and the well-established protective effects against allergic disorders related to the presence of older siblings, early colonization of this species may have profound consequences for child health.

## Abstract (precis)

*Faecalibacterium prausnitzii* has been suggested to constitute a key marker of a healthy gut, yet the factors shaping the colonization of this highly oxygen-sensitive, non-spore-forming species in the intestinal environment remain poorly understood. Here, we provide evidence from three separate infant study populations that *F. prausnitzii* colonization in the gut happens during late infancy and is affected by the number of older siblings in the family. We conclude that *Faecalibacterium* acquisition is highly likely to be accelerated by contact between siblings. Bearing in mind the immunoregulatory properties of *F. prausnitzii* and the well-established protective effects against allergic disorders related to the presence of older siblings, early colonization of this species may have profound consequences for child health.

## OBSERVATION

Starting around birth, the infant gut is colonized by a bacterial community, which gradually increases in richness and diversity (1, 2). A number of factors, including mode of delivery, breastfeeding, and introduction of complementary feeding affect this colonization. After approximately 3 years, the gut microbiota of the child is comparable to that of an adult (3).

*Faecalibacterium prausnitzii*, which is the sole species within the genus *Faecalibacterium*, is one of the most prevalent and abundant human gut microbes (4), and it has been suggested that it constitutes a marker of gut health due to its lower abundance in patients suffering from inflammatory bowel diseases compared to healthy controls and to its immunoregulatory properties (5, 6). Currently, very little is known about factors that impact the colonization of *F. prausnitzii* during early life and the putative contribution of this species to immune system development (7). Animal studies have shown that prior colonization with other microbes is necessary to obtain robust establishment of *F. prausnitzii* in germfree rodents (8, 9), and it has been suggested that depletion of oxygen in the gut or putatively another type of conditioning of the gut environment by other microbes is required for *F.  prausnitzii* to colonize the gut (7). The sensitivity of *F. prausnitzii* to low pH and bile salts explains the observation that it predominantly colonizes the distal gastrointestinal tract of humans (6), but it remains to be established how this highly oxygen-sensitive, non-spore-forming bacterium is transmitted between individuals and which factors affect its colonization process. We therefore analyzed 16S rRNA amplicon sequencing data from fecal samples originating from three previous studies of Danish infants entitled SKOT I (10), SKOT II (11), and ProbiComp (12), respectively. Comparison of *Faecalibacterium* abundance to a number of selected variables allowed identification of factors influencing colonization of *Faecalibacterium*.

**FIG 1:**
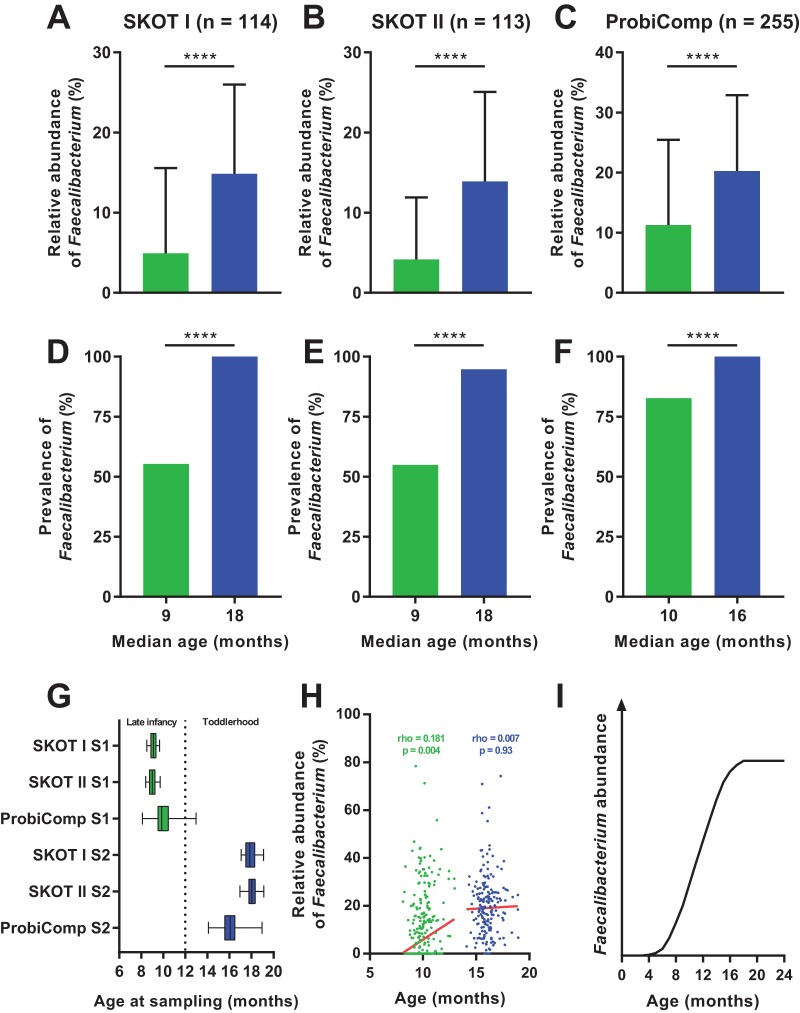
Colonization dynamics of *Faecalibacterium prausnitzii*. (A to C) Mean relative abundance of *Faecalibacterium* in SKOT I, SKOT II, and ProbiComp during late infancy (8 to 12 months old) (green) and toddlerhood (14 to 19 months old) (blue). Values are means plus standard deviations (SD) (error bars). Mean values that are significantly different (*P* < 0.0001) by Mann-Whitney test are indicated by a bar and four asterisks. (D to F) Prevalence of *Faecalibacterium* in SKOT I, SKOT II, and ProbiComp during late infancy (green) and toddlerhood (blue). Mean values that are significantly different (*P* < 0.0001) by Fisher exact test are indicated by a bar and four asterisks. (G) Variation in age at the two sampling points (sampling point 1 [S1], late infancy; S2, toddlerhood) for SKOT I, SKOT II, and ProbiComp. Boxes indicate 25th to 75th percentiles, with median values marked as a line and whiskers indicating minimum and maximum values. The dotted line indicates the age that distinguishes late infancy from toddlerhood. (H) Spearman’s rank correlations of age versus relative abundance of *Faecalibacterium* during late infancy (green) and toddlerhood (blue) in ProbiComp. Red lines indicate robust nonlinear regression fit to the data points. (I) Proposed colonization dynamics of *Faecalibacterium* as a function of age based on data from this study and previous studies (13–15).

**FIG 2:**
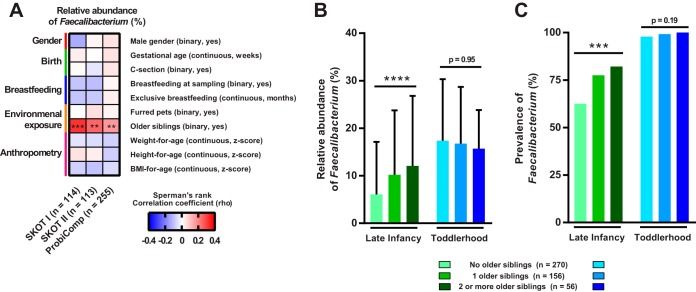
*Faecalibacterium* colonization during late infancy is impacted by older siblings. (A) Heatmap of Spearman’s rank correlation coefficients between the relative abundance of *Faecalibacterium* and selected variables during late infancy in SKOT I, SKOT II, and ProbiComp. Statistical significance is indicated by asterisks as follows: **, *P* = 0.01; ***, *P* < 0.001. (B and C) Relative abundances (B) and prevalence of *Faecalibacterium* (C) in the combined data set (*n* = 482) during late infancy and toddlerhood stratified according to the number of older siblings. Mean values plus SD (error bars) are shown in panel B. Statistical significance is indicated by asterisks as follows: ****, *P* < 0.0001 by the Kruskal-Wallis test; ***, *P* < 0.001 by the chi-square test for trend.

*F. prausnitzii* is not detected or found at very low levels during the first 4 to 6 months of life, but it increases rapidly during the first years (13–15). By investigation of three independent study populations, SKOT I (*n* = 114), SKOT II (*n* = 113), and ProbiComp (*n* = 255), we found that the relative abundance and prevalence of *Faecalibacterium* increased significantly during the period from late infancy (8 to 12 months old) to toddlerhood (14 to 19 months old) (Fig. 1A to F). In contrast to the SKOT infants, which were sampled as close as possible to 9  and 18 months of age, the infants in the ProbiComp study were sampled over a larger age span (Fig. 1G). This enabled correlation analysis between age and *Faecalibacterium* abundance and revealed a significant positive correlation at late infancy, but no such correlation at toddlerhood (Fig. 1H), suggesting that *Faecalibacterium* colonization reaches a plateau (Fig. 1I). To investigate how external factors and host variables may affect the relative abundance of *Faecalibacterium* during late infancy, we analyzed the association of its abundance with (i) gender, (ii) gestational age at birth, (iii) Caesarean section, (iv) breastfeeding, (v) pets, (vi) older siblings, and (vii) anthropometrics in all three infant study populations. Among these factors, only the presence of older siblings in the home was consistently correlated with the relative abundance of *Faecalibacterium* across the three study populations (Fig. 2A). Both the relative abundance and the prevalence of *Faecalibacterium* were higher during late infancy in infants with older siblings than in infants with no older siblings in the three populations (see Fig. S1 in the supplemental material). These differences were no longer present in toddlerhood, and *Faecalibacterium* was detected in almost all individuals (Fig. S1). Combination of the three data sets (*n* = 482) and stratification of individuals with no (*n* = 270), one (*n* = 156), or two or more (*n* = 56) older siblings revealed a stepwise increase in relative abundance (*P* < 0.0001 by the Kruskal-Wallis test) and prevalence (*P* for the trend [*P*_fortrend_] of 0.0002 by the chi-square test) of *Faecalibacterium* with increasing number of older siblings during late infancy (Fig. 2B). Moreover, age-adjusted partial correlation analysis between relative abundance of *Faecalibacterium* during infancy and the number of older siblings revealed that this association was highly significant (rho = 0.23; *P* = 6.4 × 10^−7^; *n* = 482). The association was no longer present when the children became toddlers, suggesting that at this age, children will have acquired *Faecalibacterium* from other encounters. As infants with older siblings are putatively introduced earlier to solid foods, we investigated diet as a potential confounder, since we have previously found that diet influences the microbiota (11). By analysis of detailed dietary records obtained for the SKOT cohorts (11, 16), we found that transition to family foods (rho = 0.16; *P* = 0.018; *n* = 217) and dietary intake of rye bread (rho = 0.20; *P* = 0.003; *P* value for the corrected false-discovery rate [*P*_FDRcorrected_] of 0.07; *n* = 217), but not other dietary parameters, were associated with *Faecalibacterium* (Fig. S2). However, the association between relative abundance of *Faecalibacterium* and the number of older siblings persisted after adjustment for both transition to family foods (rho = 0.26; *P* = 0.0001; *n* = 217) and intake of rye bread (rho = 0.25; *P* = 0.0001; *n* = 217). Together, this shows that rather than gender, mode of delivery, breastfeeding, complementary diet, exposure to pets or anthropometric measures, it is the exposure to older siblings that predominantly affects *Faecalibacterium* acquisition during late infancy.

10.1128/mSphere.00448-17.1 FIG S1  (A to C) Mean relative abundance (plus SD) of *Faecalibacterium* in SKOT I, SKOT II, and ProbiComp during late infancy and toddlerhood stratified according to the presence of older siblings. Statistical significance is indicated by asterisks as follows: **, *P* = 0.01; ***, *P* < 0.001 by the Mann-Whitney test. (D to F) Prevalence of *Faecalibacterium* in SKOT I, SKOT II, and ProbiComp during late infancy and toddlerhood stratified according to the presence of older siblings. Statistical significance is indicated by asterisks as follows: *, *P* < 0.05; ***, *P* < 0.001 by the Fisher exact test. Download FIG S1, EPS file, 0.02 MB. Copyright © 2017 Laursen et al. 2017 Laursen et al. This content is distributed under the terms of the Creative Commons Attribution 4.0 International license.

10.1128/mSphere.00448-17.2 FIG S2  Heatmap of Spearman’s rank correlation coefficients between relative abundance of *Faecalibacterium* during late infancy and dietary factors in SKOT I, SKOT II, and combined (SKOT I plus SKOT II). Statistical significance is indicated as follows: *, *P* < 0.05; #, FDR-corrected *P* value of 0.07. *P* values within food groups were FDR corrected for multiple testing. Download FIG S2, EPS file, 0.1 MB. Copyright © 2017 Laursen et al. 2017 Laursen et al. This content is distributed under the terms of the Creative Commons Attribution 4.0 International license.

**Study populations and samplings.** Data from three infant study populations were included in the present study. The study protocol for ProbiComp was approved by the Committees on Biomedical Research Ethics for the Capital Region of Denmark (H-4-2014-032), and the study was registered at clinicaltrials.org (NCT02180581). The study protocols for the SKOT cohorts were approved by the Committees on Biomedical Research Ethics for the Capital Region of Denmark (H-KF-2007-0003 and H-3-2010-122). All parents signed a consent form. The ProbiComp study (*n* = 290) was a double-blind randomized placebo-controlled study where infants, aged 8 to 13 months and starting daycare within 12 weeks after the start of the study were randomly assigned to receive a combination of the two probiotics, BB-12 (*Bifidobacterium animalis* subsp. *lactis* BB-12 strain) and LGG (*Lactobacillus rhamnosus* GG), or placebo during the 6-month study period (17). Inclusion criteria were single birth and starting daycare at 8 to 14 months of age. Exclusion criteria were low birth weight (<2,500 g), gestational age at birth of <36 weeks, chronic illness, regular medication, antibiotics within 4 weeks prior to the start of the study, and non-Danish speaking parents. Fecal samples were obtained before (*n* = 265; age 8 to 13 months) and after (*n* = 210; age 14 to 19 months) the study and were stored at −80°C until DNA extraction and microbiota profiling as described previously (12). The fecal microbiotas were profiled in 255 and 201 samples before and after the study, respectively, and the treatment had no impact on the fecal microbiota (12). The SKOT cohort studies monitored children during the first 3 years of life with the overall aim to investigate relationships between early diet, growth development, and later disease risks. In SKOT I (18), infants from a random sample of mothers were recruited (*n* = 311), whereas in SKOT II (19), infants of obese mothers (body mass index [BMI] of >30 kg/m^2^) were recruited (*n* = 184). Inclusion criteria for both cohorts were single birth, full-term delivery, age of 9 months ± 2 weeks at the first visit, and absence of chronic illness. Participants in both cohorts were examined at 9 (±2 weeks), 18 (±4 weeks), and 36 (±12 weeks) months of age, and fecal samples were collected and stored at −80°C until DNA extraction. The fecal microbiota data from the first two sampling points in subsets of 114 (SKOT I) and 113 (SKOT II) infants have been published previously (11).

In all study populations, fecal samples were freshly delivered on the morning of the visit or had been stored in the participant’s home, either in the freezer (−18°C) or in the fridge (4°C) for maximally 24 h before storage at −80°C. Information on gender, gestational age, Caesarean section, breastfeeding (months of breastfeeding only and the frequency of breastfeeding at sampling times), presence of older siblings and furred pets was collected from parental interviews at the sampling times in all three study populations. Anthropometrics were obtained at examinations and Z-scores were calculated as described previously (11, 17). Food records were obtained and analyzed in the SKOT studies as described previously (11, 19).

**DNA extraction and microbiota profiling.** The procedures for DNA extraction, PCR amplification, and sequencing of the V3 region of the 16S rRNA gene have been described previously (11, 12). Briefly, DNA was extracted (PowerLyzer PowerSoil DNA isolation kit [catalog no. 12855-100; MoBio]), the V3 region of the 16S rRNA gene was amplified (Phusion High-Fidelity PCR kit [catalog no. F-553L; Thermo Fisher Scientific]) with sample-specific barcoded forward primers (PBU [5′-A-adapter-TCAG-barcode-CCTACGGGAGGCAGCAG-3′]) and the universal reverse primer (PBR [5′-trP1-adapter-ATTACCGCGGCTGCTGG-3′]) according to the following PCR program: (i) 30 s at 98°C; (ii) 24 cycles, with 1 cycle consisting of 15 s at 98°C and 30 s at 72°C; (iii) 5 min at 72°C. PCR products were purified (HighPrep PCR magnetic beads [catalog no. AC-60005; MAGBIO]), DNA quantities were measured (Qubit dsDNA [double-stranded DNA] HS [high-sensitivity] assay [catalog no. Q32851; Invitrogen]), and samples were pooled to obtain equimolar libraries, which were sequenced using the Ion OneTouch and Ion PGM (personal genome machine) platform with an Ion 318 Chip kit. Sequence data were analyzed as described previously for SKOT data (11) and ProbiComp data (12), and the levels of all sequences classified as *Faecalibacterium* by the RDP classifier, with a confidence threshold of 0.5 (20) against the Greengenes database v. 13.8 (21) were pooled within each sample and normalized to the total reads in that sample.

**Statistics.** Mann-Whitney, Kruskal-Wallis, Spearman’s rank correlations, Fisher exact test, and chi-square tests were performed with the GraphPad Prims software (v. 7.0; GraphPad Software, Inc., La Jolla, CA). When indicated, *P* values were corrected for multiple testing using the false-discovery rate (FDR) (22). Partial Spearman’s rank correlation analysis adjusted for age or dietary parameters was performed in R (version 3.1.0; R Core Team. 2014. R: a language and environment for statistical computing. R Foundation for Statistical Computing, Vienna, Austria) using the *ppcor* package.

**Accession number(s).** Sequencing data are deposited at NCBI Sequence Read Archive with the accession number SRP100762 under BioProject PRJNA360073 for ProbiComp study and accession number SRP052851 under BioProject PRJNA273694 for SKOT cohorts.
